# A clinical trials corpus annotated with UMLS entities to enhance the access to evidence-based medicine

**DOI:** 10.1186/s12911-021-01395-z

**Published:** 2021-02-22

**Authors:** Leonardo Campillos-Llanos, Ana Valverde-Mateos, Adrián Capllonch-Carrión, Antonio Moreno-Sandoval

**Affiliations:** 1 Computational Linguistics Laboratory, Universidad Autónoma de Madrid, C/Francisco Tomás y Valiente 1. Cantoblanco Campus, 28049 Madrid, Spain; 2 Medical Terminology Unit, Spanish Royal Academy of Medicine., C/Arrieta 12, 28013 Madrid, Spain; 3 Complejo Asistencial Hospital Benito Menni., C/Jardines 1, 28350 Ciempozuelos, Madrid Spain

**Keywords:** Clinical Trials, Evidence-Based Medicine, Semantic Annotation, Inter-Annotator Agreement, Natural Language Processing

## Abstract

**Background:**

The large volume of medical literature makes it difficult for healthcare professionals to keep abreast of the latest studies that support Evidence-Based Medicine. Natural language processing enhances the access to relevant information, and gold standard corpora are required to improve systems. To contribute with a new dataset for this domain, we collected the Clinical Trials for Evidence-Based Medicine in Spanish (CT-EBM-SP) corpus.

**Methods:**

We annotated 1200 texts about clinical trials with entities from the Unified Medical Language System semantic groups: anatomy (ANAT), pharmacological and chemical substances (CHEM), pathologies (DISO), and lab tests, diagnostic or therapeutic procedures (PROC). We doubly annotated 10% of the corpus and measured inter-annotator agreement (IAA) using F-measure. As use case, we run medical entity recognition experiments with neural network models.

**Results:**

This resource contains 500 abstracts of journal articles about clinical trials and 700 announcements of trial protocols (292 173 tokens). We annotated 46 699 entities (13.98% are nested entities). Regarding IAA agreement, we obtained an average F-measure of 85.65% (±4.79, strict match) and 93.94% (±3.31, relaxed match). In the use case experiments, we achieved recognition results ranging from 80.28% (±00.99) to 86.74% (±00.19) of average F-measure.

**Conclusions:**

Our results show that this resource is adequate for experiments with state-of-the-art approaches to biomedical named entity recognition. It is freely distributed at: http://www.lllf.uam.es/ESP/nlpmedterm_en.html. The methods are generalizable to other languages with similar available sources.

## Background

The paradigm of Evidence-Based Medicine (EBM) [1] aims at bringing to the patient the latest research developments supported by systematic reviews and medical practice. Critical sources of evidence come from clinical trials. Nevertheless, the large volume of published information is one of the burdens for healthcare professionals to keep up to date with the latest advances. Only in 2019, 32 521 trial announcements were published on the ClinicalTrials site [2], and over 4300 in the European Union Clinical Trials Register (EudraCT) [3].

Although information retrieval techniques allow health professionals to browse the key data, queries tend to match strings. To the best of our knowledge, fine-grained search that considers the term semantics (i.e. domain classes such as *drug*, *pathology* or *procedure*) is not implemented yet. Search or information extraction systems may cluster ambiguous strings regardless of their class; e.g. *radio* may refer to a chemical element, a body part or be an abbreviation of a procedure (‘radiotherapy’). Likewise, medical professionals may have difficulties in finding information about the type of intervention they look for (e.g. pharmacological vs. surgical interventions). For example, for treating some cancers, several trials tested immunotherapy agents (experimental drugs such as nivolumab), and others, surgical or therapeutic procedures (e.g. chemohyperthermia). Access to specific types of interventions could be faster if professionals could customize their search and restrict it to chosen semantic classes. This could also help to infer relations between interventions that are potentially related or that achieve the desired outcome, which requires perusing a (frequently) large amount of evidence sources. Enriching these texts with semantics is a potential benefit to enhance the access to hidden information.

Moreover, from the patient’s viewpoint, trial announcements are written with medical terms that may not be understood. This lack of understandability hinders patients’ participation in trials. Semiautomatic text simplification techniques may alleviate this problem. To do so, biomedical named entity recognition (NER) can help to detect the candidate terms to simplify.

The objective of this work is to present the first annotated collection of texts about clinical studies and trial announcements in the Spanish language. This resource is aimed at conducting experiments for medical NER and developing systems that solve the mentioned issues. We have annotated journal abstracts about clinical trials and retrospective studies, published in PubMed and the SciELO repository, and clinical trial announcements from EudraCT. The entities belong to four semantic groups [4] from the Unified Medical Language System^®^ (UMLS) [5] concerning pathologies (DISO), anatomic entities (ANAT), biochemical or pharmacological substances (CHEM) and diagnostic or therapeutic procedures and lab tests (PROC). We focused on those four entity types as a *proof-of-concept* to assess whether the annotation and the named entity recognition task on these data yielded adequate results. The experiments here reported show that the annotation scheme and methodology provided adequate results. The current resource is freely available to the research community. In addition, the methods are generalizable to other languages with similar sources available (e.g. English, French or German).

This article begins with a literature review before explaining the methods: text selection and sources, annotation process and scheme, analysis of contents, inter-annotator agreement assessment, and use case experiments. We then report the results: count of texts and annotations, therapeutic areas covered, inter-annotator agreement, and experimental results. We discuss our outcomes before concluding. A supplementary graphical abstract summarizes the contents of this work (see Additional file 1).

## Related work

Influential corpora exist in the biomedical natural language processing (BioNLP) community, but most are available for the English language: e.g. the i2b2 corpora [6, 7], the GENIA [8], BioScope [9], CLEF [10], CRAFT [11] or DDI corpora [12]. The scarcity of resources for other languages remains a *challenge* [13]. In this section, we will focus on reviewing the corpora related to our task: texts on Evidence-Based Medicine (EBM) and Clinical Trials (CT), and BioNLP corpora in Spanish.

### EBM and CT corpora

A widely-used framework to formalize clinical trial data is the PICO model: a population or group of patients (P) with a medical problem undergoes an experimental intervention (I) concerning a standard therapy or comparator (C), with the expectation that the researched intervention will improve outcomes (O). However, corpora aimed at named entity recognition integrate entities annotated not only with PICO labels, but also with other domain labels (e.g. diseases or drugs).

One of the earliest annotated corpora of evidence-based texts is NICTA-PIBOSO [14], a collection of 1000 biomedical abstracts. With a similar approach to the work reported in [15], sentences were labeled manually with PIBOSO elements (Population, Intervention, Background, Outcome, Study Design, and Other). The team used the dataset for experiments to identify key sentences and test machine learning NER models (namely, Conditional Random Fields, CRF).

The work reported in [16] was among the first initiatives to annotate Clinical Trial Announcements (CTAs). This team annotated both CTAs (only the eligibility criteria) and clinical notes (medical entities and personal health information). The purpose was building gold standard corpora for information extraction and de-identification tasks. Texts were pre-annotated and revised manually. As far as we know, this resource is not freely available.

A different collection of EBM texts—from the *Journal of Family Practice* and excerpts from PubMed—is described in [17]. This team did not annotate medical entities but rather matched clinical questions to answers with evidence from the scientific literature. Their goal was creating a resource for automatic text summarization, evidence appraisal and clustering of answers relevant to medical questions. To create their resource, authors combined crowdsourcing, automated information extraction, and manual annotation.

The EBM-NLP corpus [18] includes almost 5000 PubMed abstracts about clinical trials. The team have a team of crowdsourcers (experts and laymen) annotate texts with PICO (Patients/Population, Interventions, Comparators and Outcomes) elements. Crowdsourcers also marked more detailed information in each category (e.g. age or pharmacological entity). This resource was developed to train machine learning (CRF) and deep learning NER models.

The Evidence Inference corpus [19] gathers more than 10 000 questions (*prompts*) paired with PubMed articles about RCTs. Medical doctors matched the prompts and the texts supporting the evidence. They also annotated the relationship between Intervention, Comparator and Outcomes: results might *significantly increase* or *significantly decrease* with regard to the comparator or show *no significant difference*. The dataset was used in machine learning experiments on evidence inference.

The work presented in [20] focused on identifying the similarity between outcomes reported in the scientific literature. To do so, this team annotated outcomes in a corpus of texts about clinical trials from PubMed Central; these data were later used to train deep learning algorithms (BERT-based models, [21]) for automatic similarity assessment.

The Evidence-Based Medicine Scientific Artefacts Semantic Similarity (EBMSASS) corpus [22] was collected reusing a subset of the NICTA-PIBOSO corpus [23]. The authors built this dataset to test approaches and measures of semantic similarity of clinical evidence in biomedical texts.

Lastly, the Chia corpus gathers annotations of patient eligibility criteria from 1000 clinical trials [24] for heterogeneous pathologies. Two medical professionals annotated entities and relationships, which can also be represented as annotation graphs to construct executable queries. Although other teams have also annotated eligibility criteria (e.g. [25, 26]; see more references in [24]), to the best of our knowledge, this is the largest freely available resource. The corpus was created for information extraction experiments and electronic phenotyping.

Not all these corpora report inter-annotator agreement values; for corpora where these were measured, agreement values ranged from Kappa values over 0.60 (*substantial agreement*) to Krippendorf’s alpha over 0.80 (*almost perfect agreement*). Table 1 summarizes the key features of the described corpora.

**Table 1:** EBM and CT corpora

Corpus	Text type and size	Annotations (count)
NICTA-PIBOSO [14]	10 000 sentences from 1000 MEDLINE abstracts	Sentences classified in the PIBOSO model: Population (812), Intervention (690), Background (2557), Outcome (4523), Study design (233) and Other (1564)
Deléger et al. [16]	52 FDA labels (96 675 tokens), 3503 clinical notes (>1M tokens) and CTAs (241 annotated with drugs, 51 793 tokens; 3000 annotated with disorders/symptoms, 647 246 tokens)	Disease and symptoms (12 388), medications and drug attributes (74 507)
EBM corpus [17]	Clinical Enquiries section from the *Journal of Family Practice*, and excerpts from PubMed	Medical questions (456), bottom-line answers (1396), justifications (3036); these are matched to 2908 abstracts
EBM-NLP [18]	5000 abstracts about clinical trials from PubMed (>1M tokens)	Entities corresponding to PICO elements (counts not reported)
Evidence Inference corpus [19]	More than 10 137 evidence questions (*prompts*) matched to 2419 PubMed articles about RCTs	Intervention results significantly increase (2428), significantly decrease (4470) or show no significant difference (3239)
EBMSASS [22]	1000 pairs of sentences of clinical evidence	Elements from the PIBOSO model (200 pairs for each class)
Koroleva et al. [20]	Sentences from clinical trial studies in PubMed Central	Outcomes: Primary (2000 sentences) and Reported (1940)
Chia [24]	1000 texts from ClinicalTrials.gov (12 409 elibility criteria)	15 entity types (41 487) and 12 different relationships (25 017)

### BioNLP corpora in Spanish

The MultiMedica corpus [27] is a multilingual (Japanese, Arabic and Spanish) collection of scientific and popularization texts from the health domain. It was prepared to conduct corpus and terminology studies and to develop a term extractor. Only Part-of-Speech (PoS) information was tagged. Because of proprietary rights, this resource is not freely available.

The MANTRA corpus [28] is a parallel collection of texts in English, French, German, Spanish and Dutch. Medline titles, drug labels from the European Medicines Agency (EMA) and patent titles were annotated with UMLS^®^ Concept Unique Identifiers (CUIs) and semantic types. Authors applied pre-annotation methods, revised manually and harmonized annotations to create this gold standard.

The IxaMedGS corpus [29] gathers 75 electronic health records (EHRs) annotated with disease and drug entities, and adverse drug reactions (ADRs) relations. After a lexicon-based pre-annotation, two pharmacology experts revised all texts. The corpus was collected for training a machine-learning-based system. To date, it is not freely accessible due to privacy issues.

The SpanishADR corpus [30] was built out from pharmacovigilance research on social media. Authors collected a database and a corpus of ADRs from ForumClinic, a patient-oriented site. Two annotators labeled drugs, effects and ADR relations in the web posts. This resource was then used to train a kernel-based method with distant supervision for relation extraction.

The DrugSemantics corpus [31] is a collection of summaries of product characteristics (SPCs). One nurse and two nursing students annotated entities of drug names and attributes (e.g. unit of measurement, dosage form, route or excipient) manually. The aim of this work was preparing a gold standard to evaluate a drug named entity classification system.

The IULA Spanish Clinical Record Corpus (SCRC) [32] gathers 3194 sentences from anonymized hospital reports. Three computational linguists annotated clinical entities (e.g. findings and procedures) and negation cues and scopes. This corpus is useful for developing text-mining and NLP systems.

A corpus from the radiology domain is presented in [33]. Two annotators (a medical student and an engineer) annotated 513 reports with clinical findings, body parts, negation, temporal terms, abbreviations and nine types of relations. As far as we know, this resource is not freely available.

The Biomedical Text Mining Unit has released several corpora ; we only mention those related to our task. For the 2nd Biomedical Abbreviation Recognition and Resolution (BARR) challenge [34], texts from PubMed and SciELO were annotated with acronyms and their expansion. For the PharmaCoNER task [35], this team prepared the Spanish Clinical Case Corpus (SPACCC) with texts from SciELO. They annotated proteins and chemical entities that can be normalized to SNOMED CT [36]. For the CODIESP challenge [37], this dataset was annotated with codes from the International Classification of Diseases, 10th edition (ICD-10). This team has also annotated cancer-related clinical cases for the CANTEMIST challenge [38].

The eHealth Discovery corpus [39] is a compilation of 1173 sentences extracted from MedlinePlus. Three experts in semantic analysis and twelve non-expert annotators labeled the sentences manually with a general semantic structure (e.g. entities and roles) and relations (e.g. is_a, or part_of). This team compiled this corpus for the TASS 2018 evaluation challenge [40].

The NUBes corpus [41] comprises 29 682 sentences from anonymized EHRs. Three linguists annotated negation and speculation and extended the IULA-SCRC resource by labeling uncertainty. Authors used NUBes to train a neural-network-based model to detect negation an uncertainty.

Lastly, the Chilean Waiting List Corpus (CWLC) [42] gathers 900 referrals from medical doctors in the Chilean healthcare system. Four medical students and doctors annotated entities, attributes and the relation *Has*. This is a gold standard for testing word-embedding-based and neural-based named entity recognizers.

**Table 2:** BioNLP corpora in Spanish

Corpus	Text type and size	Annotated entities (count)
MultiMedica [27]	Technical/popularizing texts; 4204 in Spanish, >4M tokens	No entities annotated, only part-of-speech
MANTRA corpus [28]	Multilingual; in Spanish, texts from EMA (100; 1961 tokens) & Medline (100; 1087 tokens)	UMLS semantic types and CUIs; 5530 total annotations (756 in Spanish)
IxaMedGS [29]	75 clinical reports (41 633 tokens)	Disease (2766), Drug (1191) and adverse drug reactions relations (228)
Spanish ADR [30]	397 texts from ForumClinic (26 519 tokens)	Drugs (187) and adverse drug reactions (636)
Drug Semantics [31]	30 texts from Summaries of Product Characteristics (226 729 tokens)	Disease (724), Drug (657), Measurement (557), Excipient (66), Composition (62), Dose Form (45), Route (42), Medicament (37), Food (31), Therapeutic Action (20)
IULA-SCRC [32]	3194 sentences from 300 anonymized clinical records	Body part (7), Substance (14), Finding (1064), Procedure (93), Negation (1207)
Cotik et al. [33]	513 radiology reports	Anatomy (4398), Finding (2637), Location (722), Measure (3210), Texture (1890), Measure Type (1127), Negation (1207), Uncertainty (109), Abbreviation (880), Temporal (35), Multiword (788); 9 relation types (10 987)
BARR2 [34]	3563 report cases (1 433 685 tokens)	Abbreviations, acronyms and expanded terms (9552 annotations)
SPACCC [35]	1000 clinical cases published in journals from SciELO (396 988 tokens)	PharmaCoNER: Proteins (3009), Normalizable to SNOMED CT (4398), Not-normalizable (50), Unclear (167). CODIESP: 18 483 ICD-10 codes
eHealth Discovery	1173 Spanish health-related sentences from MedlinePlus	Entities (7188), Roles (3586) and 4 types of relations (2339)
NUBes [41]	29 682 sentences from 7019 anonymized EHRs	Negation (7567 sentences) and Speculation (2219 sentences)
CWLC [42]	1912 sentences (36 157 tokens) from 900 referrals	9029 entities (Symptom, Diagnostic, Therapeutic or Laboratory Procedure, Family Member, Disease, Body part, Medication, Result, Abbreviation), 385 attributes (5 types), 284 relations

The inter-annotator agreement values of the mentioned corpora range from *moderate* to *almost perfect agreement*. However, the subset of texts doubly annotated varies from the full corpus [29] to only a 5% [35]. Table 2 shows the key features of the described resources.

## Methods

### Text sources

We downloaded 920 abstracts of clinical trial studies in Spanish, published in journals with a Creative Commons license. Most were downloaded from the SciELO repository [43], but we also resorted to free abstracts in PubMed [44]. We retrieved texts with the following query: Clinical Trial[ptyp] AND “loattrfree full text”[sb] AND “spanish”[la]. From both sources, we selected 500 texts by applying the methods explained in the section Text Selection.

We also downloaded 6021 announcements of clinical trials protocols from February to June 2020. Texts were published at the European Union Clinical Trials Register (EudraCT) and the Spanish Repository of Clinical Trials (REEC) [45]. From those texts, we only used a subset of 5272 documents; we discarded texts not available in Spanish or without the contents considered (e.g. some pediatrics texts lack a title). Following previous work [46], we were only interested in annotating the following sections: Public and Scientific Title, Public and Scientific Indication, and Inclusion and Exclusion Criteria. We finally chose 700 texts from this source. Of note, we included 52 trial protocols announcements related to the COVID-19 pandemics.

The subset of abstracts has the characteristics of formal, scientific literature aimed at specialists. Texts tend to be longer (average of 282.5±70.2 words) and contain fewer but longer sentences (7284, 14.57±4.38 average sentences per text). Besides, they have medical terms that are hard to be understood by non-health professionals. EudraCT trial announcements tend to be shorter (average of 215.61 ±69.38 words). Although they gather more sentences (13 788, 19.70±8.23 average sentences per text), these are shorter (many are list items of the eligibility criteria). These texts also feature formal, clinical writing aimed at professionals, but some sections are also written in a patient-oriented style. Namely, sections Public Title and Public Indication are generally a shorter description of the trial title and the pathology under investigation. For laymen to understand them, these sections feature simpler words and paraphrases of medical terms (e.g. *dolor postoperatorio*, ‘postoperative pain’ $\leftrightarrow$ *dolor después de la operación*, ‘pain after surgery’). Compare, for example, the following Scientific and Public Indication sections (respectively, upper and lower lines below) extracted from the CTA no 2014-000305-13:

*Prevención del tromboembolismo venoso (TEV) sintomático y la mortalidad por TEV tras el alta hospitalaria en pacientes con procesos médicos de alto riesgo* (‘Prevention of symptomatic venous thromboembolism (VTE) and VTE-related death posthospital discharge in high-risk, medically ill patients.’)

*Prevención de la aparición de un coágulo de sangre dentro de un vaso sanguíneo que bloquea el flujo de sangre a través del sistema circulatorio en pacientes que han sido dados de alta del hospital* (‘Prevent the occurrence of a blood clot inside a blood vessel that blocks the flow of blood through the circulatory system in patients who have been discharged from the hospital.’)

We found more misspellings, tokenization and mistranslations in the EudraCT subset. These errors might be due to unrevised translations and typos when registering the data in the trial register system. The editorial corrections that are mandatory for article abstracts to be published might seldom be made in CTAs.

### Text selection

We applied the methodology from [47], which is summarized herein. We distributed documents in sets of 5-6 texts each. Herein, we refer by *text* to a journal abstract or clinical trial announcement with an unique identifier (e.g. a PubMed ID or EudraCT code) and made up of several sentences. The file of each text bears the name of the corresponding identifier. First, texts were classed in percentiles according to their length: short (1st–25th percentile), medium (26th–75th percentile) and long (76th–100th percentile). Then, we sampled the texts randomly and distributed them in sets, each having one short text, one long text, and three or four medium-size texts. By applying this procedure, we tried to achieve homogeneous sets to annotate.

Second, we examined the similarity of the semantic contents. We pre-annotated the texts with the UMLS^®^ semantic groups considered (the pre-annotation is explained in section Pre-annotation of Entities). Next, we computed the distribution of semantic groups in each file—i.e. how many ANAT, CHEM, DISO or PROC entities appeared before the revision—and compared the distributions to those of each entire subcorpus. We computed distributions with the Kullback-Leibler (KL) divergence [48]. This measure describes the dissimilarity between two probability distributions, and is computed with this formula:

$$\begin{aligned} D( P \Vert \; Q) = \sum _{i=1}^{t} p_i \log \frac{p_i}{q_i} \end{aligned}$$

where P and Q are two probability distributions. The more the distributions are identical, the KL divergence is closer to zero. For each set of 5-6 files, we computed the KL value, compared it to those of the entire subcorpus (abstracts or EudraCT) and sorted sets in increasing order, selecting only the needed sets. With this procedure, we chose the sets with the smallest KL value—i.e. the texts with the most similar distribution to each subcorpus.

Finally, when we had annotated 1000 texts, we decided to enlarge the corpus with 200 documents. We again applied the previous methods to choose the last batches to annotate, but also the suggestions to select training data for NER tasks, provided in a very recent work we found [49] after having annotated 1000 texts. These authors compared several measures, namely the vocabulary shared between texts, the language model perplexity or the word vector variance; overall, these authors reported that each measure had a similar predictive value. Therefore, we computed the vocabulary shared between candidate texts and the 1000 texts already in the corpus. We finally selected the texts with the higher similarity values of vocabulary with regard to the 1000 documents already included in the dataset.

In domains where publicly available data are scarce, a text selection method is critical to build a corpus with an adequate size and enough generalizable data. If enough sources are available, gathering large volumes of data might suffice; however, experiments in the medical domain have already shown that larger datasets do not necessarily yield better results [50]. This is the reason why we selected texts according to their similar length or semantic content (by applying the KL distance on the semantic annotations) and the lexical similarity (Dai et al.’s method [49]). For our task, these methods are complementary and are more adequate than other alternatives such as selecting texts according to the authors’ demographics or the publication channel (e.g. forum posts vs. scientific/regulatory agencies platforms).

### Analysis of corpus contents

We analyzed qualitatively the therapeutic areas covered in the trial studies and announcements. We counted the texts according to the Medical Subject Heading (MeSH) Tree Entry Term that could best describe them. For the texts from EudraCT, we took the class in the trial announcement (section E.1.1.2). For the abstracts, we did not have this information available. We classified the texts manually by considering the MeSH descriptors that journals had assigned to the abstracts in PubMed or SciELO, and the type of journal where they were published. Note that this approach is less accurate than the classification of texts from EudratCT. However, descriptors from EudraCT do not always describe the texts accurately, and some medical conditions can be categorized into several classes: e.g. texts about COVID-19 are classed into C2 Virus Diseases, but sometimes are classed into C08 Respiratory Tract Diseases. We nevertheless followed the classification from EudraCT. Consequently, because of the above reasons, this analysis should be taken with caution; it is only an overall view of what our corpus covers.

### Pre-annotation of entities

We pre-annotated the data to speed up the annotation, given that some research teams [46] obtained optimal results without annotation biases. We applied a hybrid named entity recognition pipeline, implemented in Python and spaCy [51]. The NER pipeline is made up of a module for dictionary-based matching, normalization, tokenization and lemmatization. Post-processing rules are used to exclude specific UMLS^®^ semantic groups (e.g. CONC, GENE or PHYS groups were not annotated in the current version). Rules of term composition widen the coverage of annotated entities (e.g. *enfermedad de* + proper name $\rightarrow$ DISO; e.g. *enfermedad de Crohn*, ‘Crohn’s disease’). We used MedLexSp [52], a Spanish lexicon with terms from most medical terminologies and knowledge bases: e.g. ICD-10, MeSH, SNOMED CT or the *Dictionary of Medical Terms* [53]. A supplementary video shows the interface of the tool for the preannotation (see Additional file 2).

### Annotation scheme

This version of the corpus is aimed at experiments on named entity recognition. We annotated four types of entities corresponding to UMLS^®^ [5] semantic groups (SG) of pathologies (DISO), anatomic entities (ANAT), biochemical or pharmacological substances (CHEM) and lab tests, diagnostic or therapeutic procedures (PROC). For a first version of the corpus, and given the budget and time constraints, we focused on the most relevant subset of UMLS groups for the task. Table 3 shows the list of annotated SGs, the correspondence to UMLS^®^ semantic types, and examples.

**Table 3:** Annotated UMLS^®^ semantic groups (SG) and semantic types, with examples

SG	Semantic types	Examples
ANAT	Anatomical structure; body location or region; body part organ or organ component; body space or junction; body substance; body system; cell component; cell; embryonic structure; fully formed anatomical structure; tissue	*Sangre* (‘blood’), *músculo* (‘muscle’)
CHEM	Amino acid, peptide, or protein; antibiotic; biologically active substance; carbohydrates; chemical; chemical viewed functionally; chemical viewed structurally; clinical drug; element, ion, or isotope; enzyme; hazardous or poisonous substance; hormone; immunologic factor; indicator, reagent, or diagnostic aid; inorganic chemical; nucleic acid, nucleoside, or nucleotide; organic chemical; pharmacological substance; receptor; vitamin	*ADN* (‘DNA’), *antibiótico* (‘antibiotic’), *penicilina* (‘penicillin’), *tacrolimus*, *retinol*, *calcio* (‘calcium’)
DISO	Acquired abnormality; anatomical abnormality; cell or molecular dysfunction; congenital abnormality; disease or syndrome; experimental model of disease; injury or poisoning; mental or behavioural dysfunction; pathologic function; neoplastic process; sign or symptom	*Cancer*, *diabetes*, *fiebre* (‘fever’), *mutación* (‘mutation’)
PROC	Diagnostic procedure; health care activity; laboratory procedure; molecular biology research technique; research activity; therapeutic or preventive procedure	*Hemograma* (‘hemogram’), *diálisis* (‘dialysis’)

Note that we annotated all these types of entities, regardless of whether they occurred in negated contexts or not. For example, *ostomía* (‘ostomy’) is annotated in *sin ostomía* (‘without ostomy’). Qualifiers or modifiers were only annotated as part of a broader entity (and with the same label) provided that the full entity could be normalized to a reference terminology or code. For example, *crónica* (‘chronic’) was not annotated as concept (CONC) in *enfermedad renal crónica* (‘chronic kidney disease’); we rather annotated *enfermedad renal crónica* as DISO, because this entity can be normalized to an ICD-10 code (N18.9) or UMLS CUI (C1561643). We did not annotate discontinuous nor overlapping entity mentions.

To design the annotation scheme, we reviewed the guidelines of available corpora [6, 10, 12, 28, 29, 31, 35, 47]. We also considered annotating PICO elements (Patients/Population, Interventions, Comparators, and Outcomes) instead of UMLS^®^ groups. We nevertheless discarded annotating PICO elements in this version of the corpus, given the need for several annotators with expert knowledge and medical background to carry out this type of annotation. We also chose to annotate UMLS groups because we did not want to restrict the utility of our corpus to process only clinical trials. Our goal was to release a resource that could help to process also other broader medical text sources that support Evidence-Based Medicine and are not formalized with the PICO framework (e.g. clinical practice guidelines and, to some extent, medical records).

Because we first aimed at building a NER corpus, we did not conduct a systematic concept annotation and normalization to reference terminologies or ontologies as in the CRAFT [11] or MANTRA corpora [28]. Systems such as MetaMap [54] provide automatic UMLS concept recognition; however, concept normalization requires manual revision and considerably deeper disambiguation and time investment. Although our choice limits the utility of the corpus, we nonetheless added a small fraction of CUIs manually during the annotation process for understanding the labeled entities. In addition, we thought it beneficial to add at least those CUIs that could be mapped automatically to the annotated entities. We used exact string matching and the MedLexSp lexicon [52] to add only those CUIs that matched our annotations (changed to lowercase) and corresponded to the semantic group we annotated. This was required to avoid assigning a wrong CUI to ambiguous strings. For example, *calcio* was matched to C0006675 when referring to the chemical element (CHEM); but it was matched to C0201925 when referring to the laboratory procedure (PROC). In multi-word entities, the full entity was matched (not parts of them): e.g. in *calcio sérico* (‘serum calcium measurement’, C0728876), the CUI does not refer to *calcio* nor to *sérico*. Note that this procedure has limitations and not all the annotations are normalized automatically to CUIs. For example, we could not normalize some derived forms (*lobar* $\leftrightarrow$ *lóbulo*, ‘lobe’, C0796494), shortened forms (*sd de malabsorción* $\leftrightarrow$ *síndrome de malabsorción*, ‘malabsorption syndrome’, C0024523), paraphrases (*asignados al azar* $\leftrightarrow$ *aleatorizados*, ‘randomized’, C0034656) or misspellings (*cromosopatía, ‘chromosomopathy’, C0008626). Therefore, the normalized annotations are of limited utility for evaluating how concept recognition systems deal with linguistic variability in these texts. On the other hand, the amount of CUIs provided, to the best of our knowledge, outnumbers the data in other Spanish corpora, and builds the foundations for future annotations.

### Annotation process

**Fig. 1:**
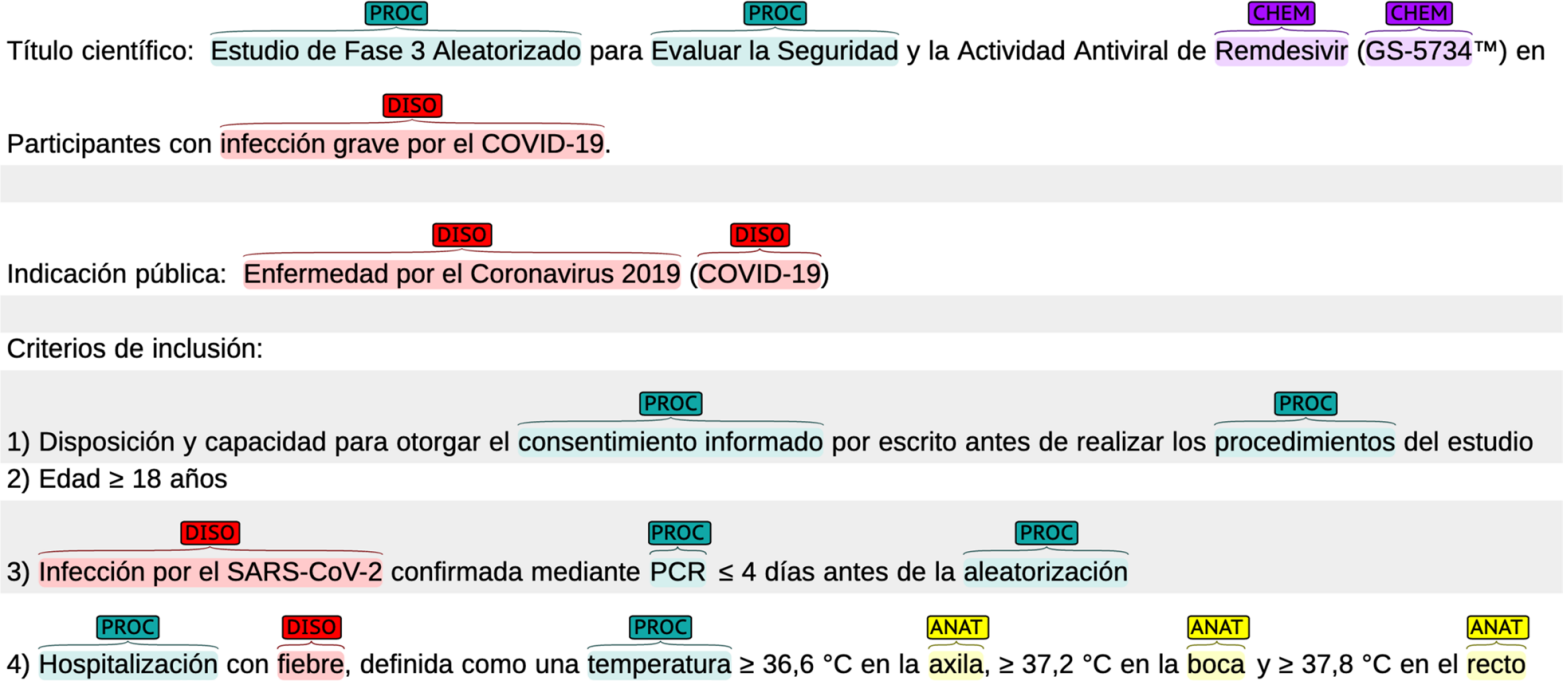
Sample of the annotation

**Fig. 2:**
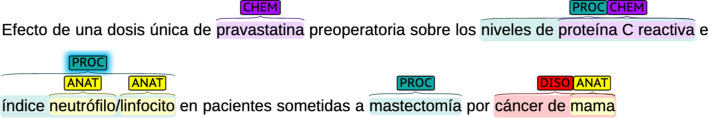
Sample of nested annotations

We used the BRAT Rapid Annotation Tool [55] for the annotation; Fig. 1 shows a sample. Note that we also annotated nested entities [56]; for example, both a disease or procedure and the affected body part(s) are marked. Figure 2 shows nested entities: e.g. *cáncer de mama* (‘breast cancer’) is annotated as DISO and includes the annotation of *pecho* (‘breast’) as ANAT.

**Fig. 3:**
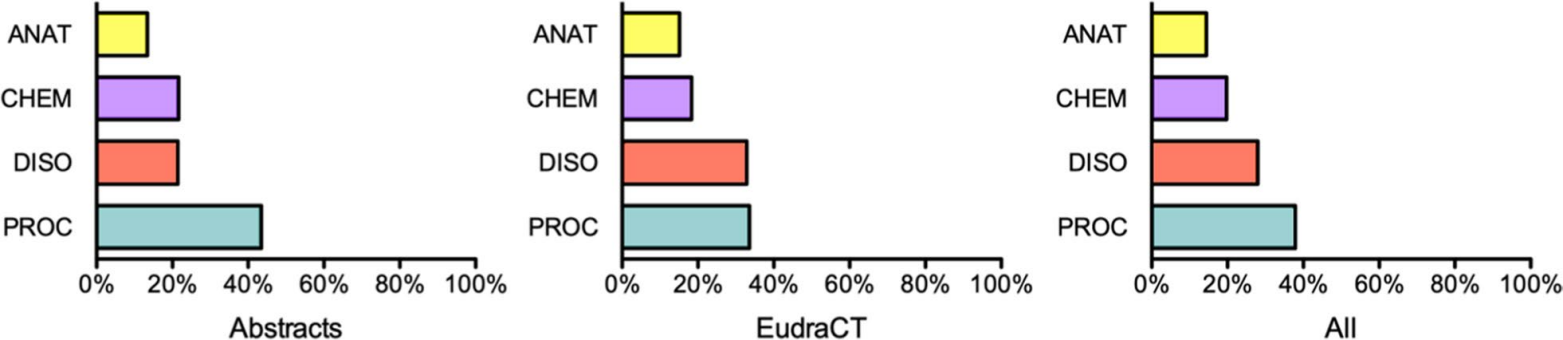
Distribution of annotated entity types (in percentage)

**Fig. 4:**
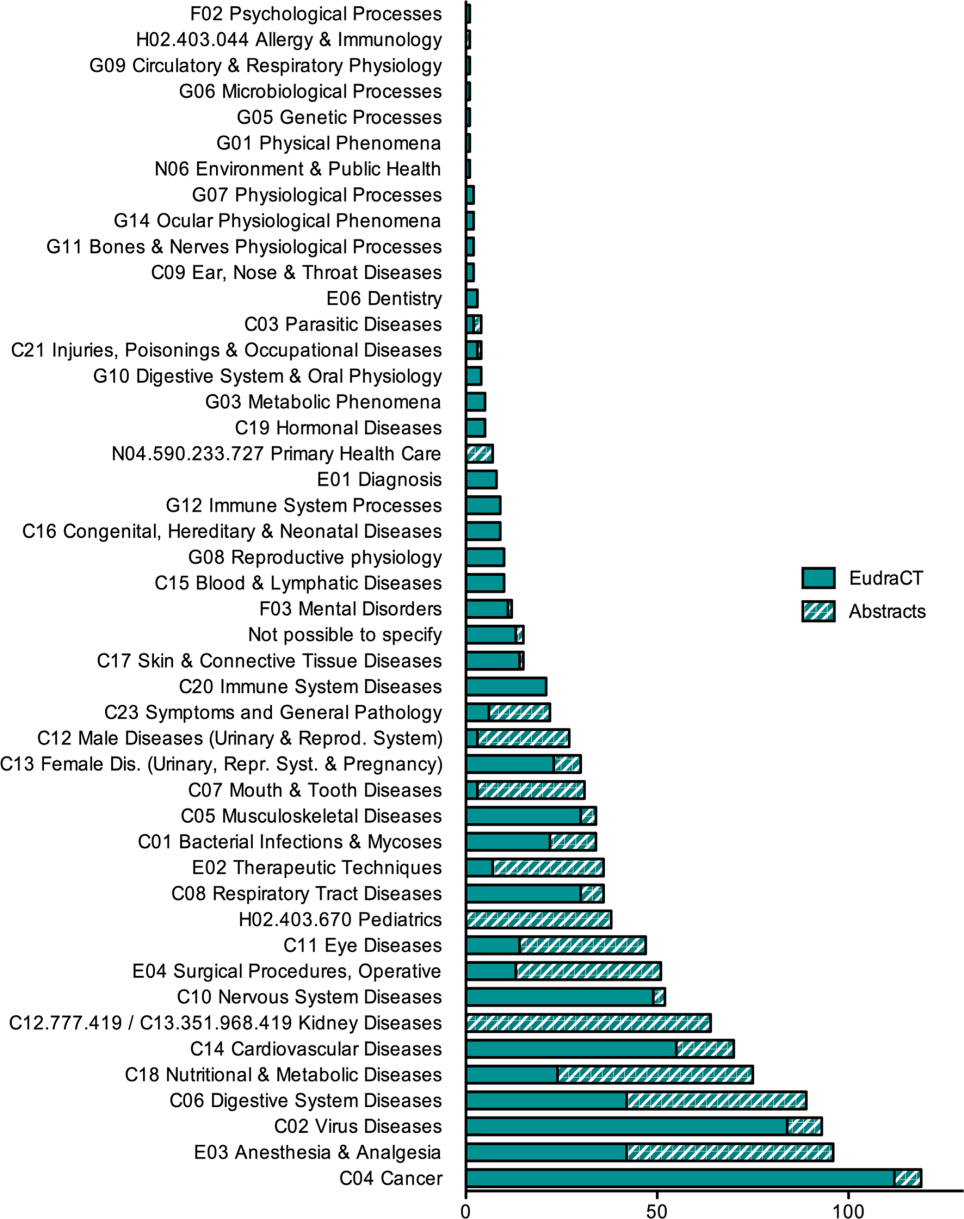
Therapeutic areas of texts (codes correspond to MeSH tree numbers)

Three researchers (co-authors of this work) were involved in the task: a medical practitioner (ACC), a medical terminologist (AVM), and a computational linguist (LCL), who coordinated the annotation task and normalized all the annotations. The annotation process was conducted in three stages. In the first stage, all annotators (*triple annotation*) labeled the same documents (12 abstracts). The triple annotation was a means of *training* all three annotators using the same texts and discussing and modifying the annotation criteria among all participants. After meetings to fix the annotation criteria, we set up consensus annotations and computed the inter-annotator agreement. Once we saw that the IAA value was adequate, we fixed a first version of the annotation guidelines. We then proceeded to the second stage (*double annotation*): since the three annotators could not revise the same documents because of time constraints, a pair of annotators doubly revised a subset of 49 texts, and another pair revised a different sample of 63 texts. In total, 112 texts were doubly annotated to compute the inter-annotator agreement. We first doubly annotated the journal abstracts, then the clinical trial announcements from EudraCT. The three annotators held meetings to achieve consensus annotations regularly every one or two weeks. During this process, the annotation guidelines were fixed and updated on a regular basis. The final annotation guidelines are available at the project web site.^1^ The last stage of the annotation (*harmonization*) was carried out after all texts were annotated. The coordinator of the annotation task unified and suppressed incoherent annotations across all documents. The full process lasted over seven months.

### Inter-annotation agreement (IAA)

To measure the annotation quality, we computed the IAA for 124 files (approximately, 10% of the data). Around two-thirds of the texts (67%) for measuring the IAA were chosen randomly, whereas one-third of texts were chosen due to specific difficulties we wanted to solve (in particular, by the medical doctor). We could not doubly annotate more documents owing to time and budget constraints.

We calculated the inter-annotator agreement through the F-measure value. We did not use the Kappa value because entity spans were also compared, which can be problematic since the expected chance agreement of each entity type and span can be extremely scarce [57]. Nonetheless, in annotation contexts where entities might have different spans (e.g. *hepatitis* or *hepatitis grave*, ‘severe hepatitis’), it is adequate to use the F-measure as a measurement of agreement between one set of annotations and the other doubly annotated set (taken as the reference) [58].

### Use case

To determine the validity of the CT-EBM-SP corpus and present a real use case, we report experiments using this resource in the context of a supervised named entity recognition (NER) task. Note that the goal is not to compare current NER approaches systematically, nor to test the latest neural architectures that are out of reach of our computational resources (e.g. GPT3 [59]). We rather intend to set a tentative baseline with this corpus and show that this first version is adequate for testing models. We tested three frameworks based on a language-modeling objective, given that this yields better results for NER than the classic embedding approaches [60, 61]. In the following, we describe the algorithms, the methodology and the evaluation procedure.

#### SequenceLabeler

We first tested SequenceLabeler [62], a neural-based sequence labeling architecture. It is a Bidirectional Long-Short Term Memory (Bi-LSTM) model with a final layer implementing Conditional Random Fields (CRF); this is similar to the framework proposed in [63, 64]. SequenceLabeler also computes a language model and trains character embeddings along with token embeddings, applying an attention mechanism. Out-of-Vocabulary (OOV) words are replaced with the UNK token. This framework has achieved competitive results in supervised tasks such as learner error detection, named entity recognition or PoS-tagging.

We trained our own medical word-embeddings with fastText [65] and used the same hyperparameters of the article [62]: dimension of tokens = 100, dimension of characters = 50, Adadelta optimizer, learning rate = 1, dropout = 0.5, batch size = 64, and minimal word frequency = 1. Character tokens were not lowercased. We set the training to a maximum of 50 epochs (although we did not achieved that maximum); the training stopped if the model did not improve after 7 epochs of evaluation on the development set.

#### Contextual string embeddings (Flair)

We also tested a Bi-LSTM-CRF architecture using contextual string embeddings provided in the Flair framework [66]. Contextual string embeddings represent words as sequences of characters contextualized by the surrounded text. For each word, the internal states of a bidirectional character-level language model are retrieved. Both forward and backward representations can be stacked with pre-trained word-level embeddings. The stacked embeddings are input to a Bi-LSTM-CRF module to predict the labels. Flair features several pre-trained language models, embeddings and functions to stack different language representations.

We stacked the medical fastText embeddings (the same employed with SequenceLabeler) and the contextual string embeddings provided in Flair; these are general embeddings pre-trained using the Spanish Wikipedia. We applied almost the same hyperparameters as in [66]: stochastic gradient descent optimizer, hidden states per layer = 256, dropout = 0.5, and batch size = 32. Likewise, the learning rate was initialized to 0.1, and halved if training loss did not improved for 5 epochs. The maximum number of epochs was set to 100 (although our experiments stopped training before that limit). We provide a Python notebook for replicating the experiment.

#### Bidirectional encoder representations from transformers (BERT)

Bidirectional Encoder Representations from Transformers (BERT) [21] is a language representation model featuring contextualized embeddings. It is trained with self-attention layers of the Transformer encoder [67] and a masked language model (MLM), which replaces randomly 15% of input tokens with a mask token. The training objective is to predict the original replaced word; this enables pre-training both the right and left context. The BERT framework uses WordPiece embeddings and the UNK token replaces Out-of-Vocabulary (OOV) words. BERT involves two steps: unsupervised *pre-training*, and *fine-tuning* the pre-trained representations for a supervised task. For the first step, the standard English BERT model was trained in BooksCorpus (800M words) and Wikipedia (2500M words).

We tested a BERT model for Spanish (BETO) [68]. BETO was pre-trained on several corpora (3000M tokens), including the Spanish versions of Wikipedia, EMA, EuroParl or News-Commentary vs 11. We used the BERT base model trained on 12 layers, with a hidden size of 768 and 12 attention heads. The learning rate was 3e-5, using the Adam optimizer, and tokens were not lowercased. The batch size was 8, and the sentence length was 270 (we padded shorter sentences to fit that length). For the fine-tuning step, we plugged a layer for named entity recognition (without Conditional Random Fields) on top of the Spanish BERT. We implemented it in PyTorch with the Transformers library [69]. We trained for 4 epochs, as in the BERT paper [21]. We make available a Python notebook with the code for the replicability of results.

#### Experiment methods

The procedure followed a standard methodology. The annotated files in BRAT format were converted to the CoNLL tabular format, and entity types were formatted with the Begin (B), Inside (I) and Out (O) scheme. In preliminary tests, we also tested the BIOES format (where *E* stands for ‘End’, and *S*, for ‘single’), since other researchers reported higher results [70]. However, we did not use it finally because the improvements were not substantial.

We trained all neural frameworks on a corpus subset (60%) of 720 texts (175 203 tokens): 300 abstracts and 420 texts from EudraCT. We validated the model on a development set (20% of the corpus) of 240 texts (58 670 tokens; 100 abstracts and 140 EudraCT announcements). Lastly, we tested the best configuration of each model on a 20% of the corpus (240 texts, 58 300 tokens), with the same distribution as in the development set (see Table 8 in Results). We used an NVIDIA GeForce RTX 2080 TI Turbo 11GC to train the BERT NER and Flair models.

For SequenceLabeler and Flair, we used fastText word-embeddings [65]. We trained them on Spanish texts of the medical domain from the European Medicines Agency corpus [71] ($\sim$13.9M tokens) and articles from the SciELO repository ($\sim$25M tokens). The vocabulary size is of 61 752 tokens. We applied the following parameters: Skip-gram model, window size = 10, dimensions = 100, minimum frequency = 1, number of negatives sampled = 10, learning rate = 1e-4. The embeddings can be downloaded at the project website.

#### Evaluation procedure

We computed standard precision, recall and F1 measure. Precision (P), which is also referred to as *positive predictive value*, is computed based on the count of true positives (TP) and false positives (FP):

$$\begin{aligned} P = \frac{TP + FP}{TP} \end{aligned}$$

Recall (R), also called *sensitivity*, is calculated out from the number of true positives (TP) and false negatives (FN):

$$\begin{aligned} R = \frac{TP + FN}{TP} \end{aligned}$$

Lastly, the F1 measure is the balanced ratio between P and R, and is appropriate when evaluating tasks with several unbalanced labels:

$$\begin{aligned} F = \frac{ 2 P R }{ P + R } \end{aligned}$$

We report micro-average F1 scores (strict match). We ran 10 experimental rounds with different random seeds (for training SequenceLabeler) or different random initialization of the training set (for BERT NER and Flair). We report the average precision, recall and F measures with their standard deviation.

## Results

### Descriptive statistics and count of annotations

We annotated 1200 texts to be distributed for research. One subset is made up of 500 summaries of clinical trial studies published in journals with a Creative Commons license. The other subset includes 700 announcements of clinical trials protocols, published at the European Union Clinical Trials Register (EudraCT) [3] and the Spanish Repository of Clinical Trials (REEC) [45].

Table 4 presents the counts of sentences, tokens and annotated entities in each subcorpora. We counted as sentence any text segment between sentence-boundary characters (*?, !, .*) and new lines. We did not annotate some sentences where no entity of the considered UMLS groups occurred. For example, some sentences only report the CT registration number, which we did not annotate: e.g. *Registrado en U.S. National Institutes of Health, ClinicalTrials.gov con número NCT03239808* (‘Registered at the U.S. National Institutes of Health, ClinicalTrials.gov under the number NCT03239808’). Table 5 shows the distribution per entity type; and Fig. 3, the distribution in percentage. *M* stands for ‘mean’, and *SD*, for ‘standard deviation’. PROC and DISO entities outnumber the rest of entity types. A total of 13.98% of annotations are nested. Regarding the normalization of entities, an average of 70.68% were normalized to UMLS CUIs, out of which 2088 (4.47% of annotations) were added and revised manually. For comparison, Table 6 shows counts of the pre-annotation (before revision). The number of entities decreased in the revised version, but the proportion across labels was similar to the pre-annotated data. Although the pre-annotation made it easier for annotators to detect the desired entities, it created false positives or mismatches that needed subsequent revision.

**Table 4:** Count of sentences, tokens and annotated entities

	Abstracts	EudraCT	Total
Texts	500	700	1200
Sentences	7160	11 995	19 155
M (SD)	14.32 (±4.24)	17.14 (±5.24)	15.96 (±5.04)
Annotated sentences	5444	8607	
M (SD)	10.89 (±3.00)	12.29 (±4.63)	11.71 (±4.09)
Tokens	141 245	150 928	292 173
M (SD)	282.49 (±70.21)	215.61 (±69.38)	243.48 (±77.11)
Entities	20 031	26 668	46 699
M (SD)	40.06 (±13.67)	38.10 (±14.39)	38.92 (±14.12)
Nested entities	2613 (13.04%)	3914 (14.68%)	6527 (13.98%)
Normalized	13 627	19 382	33 009
to UMLS CUIs	(68.03%)	(72.68%)	(70.68%)

**Table 5:** Distribution of annotations per entity type (*A*: ‘Abstracts’; *E*: ‘EudraCT’)

	A	M (SD)	E	M (SD)	Total	M (SD)
ANAT	2683	5.37 (±4.90)	4045	5.78 (±4.74)	6728	5.61 (±4.81)
CHEM	4338	8.68 (±7.19)	4886	6.98 (±5.07)	9224	7.69 (±6.10)
DISO	4296	8.59 (±7.20)	8771	12.53 (±6.40)	13 067	10.89 (±6.30)
PROC	8714	17.43 (±7.74)	8966	12.81 (±5.87)	17 680	14.73 (±7.09)

**Table 6:** Counts of pre-annotated entities

	Abstracts	EudraCT	Total
All	25 265	31 078	56 343
M (SD)	50.53 (±16.49)	44.40 (±16.73)	46.95 (±16.90)
ANAT	3653	4847	8500
M (SD)	7.31 (±5.21)	6.92 (±5.44)	7.08 (±5.34)
CHEM	4956	5132	10 088
M (SD)	9.91 (±7.97)	7.33 (±5.04)	8.41 (±6.55)
DISO	6555	10 732	17 287
M (SD)	13.11 (±6.59)	15.33 (±7.60)	14.41 (±7.27)
PROC	10 101	10 367	20 468
M (SD)	20.20 (±7.73)	14.81 (±6.42)	17.06 (±7.48)

### Therapeutic areas covered

Figure 4 shows our analysis. The corpus abounds with texts related to the following therapeutic areas: cancer, anesthetic procedures, virus diseases (e.g. HIV and COVID-19), digestive system diseases (e.g. Crohn’s disease), nutritional and metabolic diseases (e.g. diabetes) and kidney diseases.

### Results of the inter-annotator agreement

The average F-measure is 85.65% with a standard deviation of ±4.79 (strict), and F-measure of 93.94% (±3.31) (relaxed). These figures are average values after consensus annotations were achieved between all annotators. Following [31], we estimate that our average F-measure in the Landis & Koch scale [72] could correspond to F $\in$ [100-80] (*almost perfect agreement*). According to each stage, the inter-annotator agreement is as shown in Table 7.

**Table 7:** InterAnnotator agreement

	Texts	Mean (Standard deviation)	
		Strict	Relaxed
Triple revision	12 (abstracts)	77.0% (±4.2)	86.10% (±3.2)
Double revision	42 (abstracts)	82.62% (±2.11)	93.06% (±0.97)
	70 (EudraCT)	88.48% (±3.05)	95.61% (±1.68)
	112 (abst. + EudraCT)	86.52% (±3.92)	94.76% (±1.91)
All ($\sim$10% of texts)	124	85.65% (±4.79)	93.94% (±3.31)

If we analyze the IAA value according to the text type, we see higher IAA values in texts from EudraCT. However, these figures are not comparable, given that we first annotated the abstracts, then annotated the trial announcements. The higher values obtained could both be due to the fact that the announcements were easier to annotate, and also because we annotated these data in the last annotation stage (when annotators were fully trained). Notwithstanding this, we do see a steady increase in IAA values from the training stage (average F = 77.0% ±4.2, strict; and average F = 86.10% ±3.2, relaxed) to the last stage (F = 86.52% ±3.92, average of strict IAA for both abstracts and EudraCT; and average F = 94.76% ±1.91, relaxed). Annotators progressed steadily as they annotated more data and criteria were automated or learnt.

**Fig. 5:**
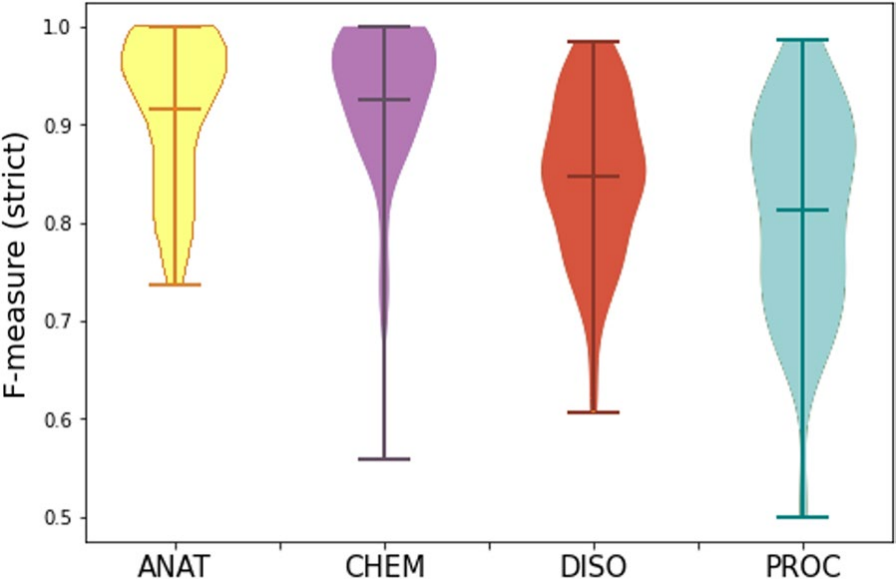
IAA per entity type (strict)

**Fig. 6:**
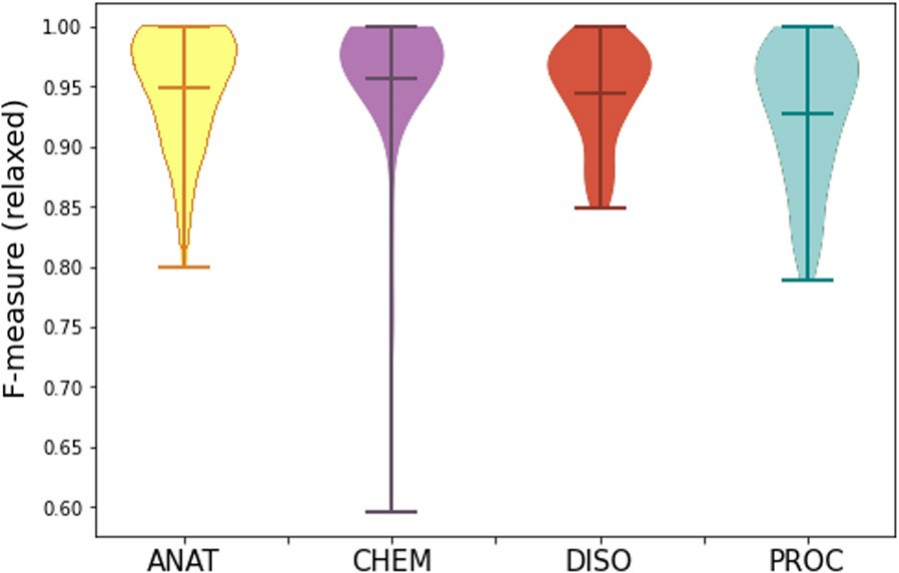
IAA per entity type (relaxed)

**Fig. 7:**
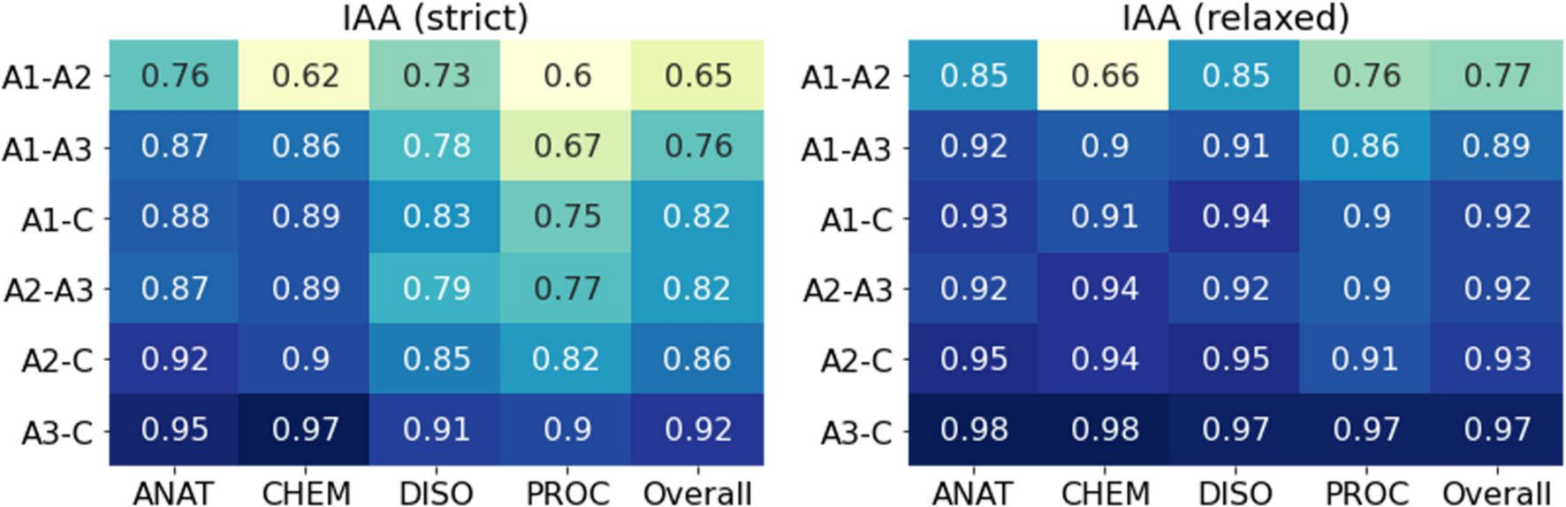
IAA values per pair of annotators and with regard to consensus (C) annotations

Figures 5 and 6 show the IAA values per entity type, and Fig. 7, IAA per pair of annotators and with regard to the consensus (C). In the strict evaluation, more disagreements between annotators concerned the PROC category, followed by the DISO label. Indeed, many differences involved the scope of the annotation, namely modifiers of multi-word terms.

### Results of the experiments

58 300

**Table 8:** Distribution of tokens (upper rows) and entities (inferior rows) per split

TOKENS	Train	Dev	Test
Abstracts	84 855	27 957	28 433
M (SD)	282.85 (±67.66)	279.57 (±56.34)	284.33 (±88.49)
EudraCT	90 348	30 713	29 867
M (SD)	215.11 (±66.93)	219.38 (±68.04)	213.34 (±77.81)
All	175 203	58 670	
M (SD)	243.34 (±75.04)	244.46 (±69.94)	242.92 (±89.41)
**ENTITIES**	**Train**	**Dev**	**Test**
Abstracts	12 129	4092	3810
M (SD)	40.43 (±13.29)	40.92 (±13.78)	38.10 (±14.63)
EudraCT	15 972	5537	5159
M (SD)	38.03 (±14.10)	39.55 (±14.70)	36.85 (±14.90)
All	28 101	9629	8969
M (SD)	39.03 (±13.81)	40.12 (±14.31)	37.37 (±14.77)
ANAT	4023	1442	1263
M (SD)	5.59 (±4.88)	6.01 (±4.78)	5.26 (±4.61)
CHEM	5577	1840	1807
M (SD)	7.75 (±6.00)	7.67 (±6.01)	7.53 (±6.50)
DISO	7832	2716	2519
M (SD)	10.88 (±6.18)	11.32 (±6.94)	10.50 (±6,01)
PROC	10 669	3631	3380
M (SD)	14.82 (±6.91)	15.13 (±7.41)	14.08 (±7.27)

We trained on 60% of the corpus and 20% for development and 20% for testing (Table 8). In the 10 experimental rounds, we trained SequenceLabeler for an average of 26.9 epochs (±5.78); and Flair, for an average of 86.20 epochs (±9.62). We trained the BERT NER model for 4 epochs, as in the original paper [21]); substantial improvements were not achieved at the 4th epoch, but the development loss had increased steadily. Tables 9 and 10 present our results.

**Table 9:** Average (±standard deviation) P, R and F1 in development and test

		Precision	Recall	F-measure
SequenceLabeler	Dev	81.02 (±1.17)	78.65 (±1.89)	79.80 (±0.82)
	Test	80.67 (±1.36)	79.91 (±1.51)	80.28 (±0.99)
Flair	Dev	82.65 (±0.35)	83.18 (±0.44)	82.92 (±0.38)
	Test	82.35 (±0.28)	83.18 (±0.29)	82.76 (±0.24)
BERT NER	Dev	88.03 (±0.27)	86.06 (±0.24)	87.03 (±0.21)
	Test	87.93 (±0.22)	85.58 (±0.31)	86.74 (±0.19)

**Table 10:** Average P, R and F1 (±standard deviation) per entity type (test set)

SeqLabeler		Precision	Recall	F-measure
	ANAT	62.32 (±6.27)	56.74 (±4.48)	59.06 (±2.27)
	CHEM	85.81 (±2.47)	82.94 (±1.84)	84.32 (±1.29)
	DISO	82.35 (±1.55)	81.11 (±1.69)	81.70 (±0.91)
	PROC	78.94 (±1.98)	79.91 (±1.72)	79.40 (±1.25)
Flair	ANAT	71.10 (±2.62)	62.25 (±1.24)	66.36 (±1.63)
	CHEM	85.98 (±0.62)	87.30 (±0.34)	86.63 (±0.33)
	DISO	84.68 (±0.29)	85.04 (±0.29)	84.86 (±0.13)
	PROC	79.90 (±0.36)	81.86 (±0.37)	80.86 (±0.27)
BERT	ANAT	63.38 (±2.14)	63.88 (±2.61)	63.56 (±1.08)
	CHEM	91.47 (±0.50)	90.74 (±0.51)	91.10 (±0.36)
	DISO	90.23 (±0.26)	88.43 (± 0.56)	89.32 (±0.23)
	PROC	85.20 (±0.43)	80.87 (±0.54)	82.98 (±0.30)

#### Error analysis

An error analysis is necessary to understand the output of the neural models, which operate as a *blackbox*. This procedure aims at helping to achieve explainable artificial intelligence systems that can be considered reliable and trustworthy—especially by medical professionals [73]. We thus analyzed the system predictions on the test set and found several errors due to ambiguous entity types. Some errors come from homonymy or polysemy: e.g. *miembro* may refer to ‘member’ (a person in a group) or ‘limb’ (anatomic entity). Besides, ambiguity affects at the semantic group. Ambiguity is very frequent among chemical entities, which often refer to the laboratory procedure measuring a substance. For example, *calcium* was annotated chem in the context of *suplementos con calcio* (‘calcium supplements’); but we labeled it as proc in *niveles de calcio sérico* in contexts where it implies *serum calcium measurement*. All neural models made errors in some of these contexts.

Other errors are due to entities with low frequency in the corpus, especially those occurring just once. The task type has an impact on this distribution of data, where some terms have low frequency. Texts from trials report experimental drugs, which occasionally do not appear in terminological resources—not even in drug databases such as DrugBank or PubChem. Similarly, trials conducted on rare or uncommon diseases have vocabulary items that can yield recognizing errors. Several acronyms or abbreviations with low frequency in the corpus also caused errors. Interestingly, vice versa, some proper names (e.g. from institutions or trial titles) caused false positives—the algorithm annotated them incorrectly in spite of its low frequency.

Other errors are related to the annotation scope. This is particularly common in adjectives of severity or degree (e.g. *grave*, ‘severe’, or *leve*, ‘mild’), and modifiers of procedures that specify the manner or details about the methods applied (e.g. *ambulatorio*, ‘ambulatory’). All neural models made errors in certain contexts (e.g. *cirugíia ginecológica abierta*, ‘open gynecologic surgery’). Annotators indeed hesitated regularly about the scope of these terms. The scope of entities to annotate may change subject to different tasks such as normalizing to a reference thesaurus, annotating detailed clinical mentions, or mapping entities to PICO elements.

**Table 11:** Examples of errors and predictions of each neural model (*B*: BERT; *F*: Flair; *SL*: SequenceLabeler)

Type	Example	Model
Ambiguity	*el nivel de fósforo se redujo* (‘phosphorus level decreased’)	
Prediction:	O B-PROC I-PROC I-PROC O O	✗: B, F, SL
Reference:	O O O B-CHEM O O	
	*grupos de 20 miembros* (‘20-member groups’)	
Prediction:	O O O B-ANAT	✗: SL
Reference:	O O O O	✓: B, F
FNs	*Lurbinectedin ( PM01183 )* (‘Lurbinectedin (PM01183)’)	
Prediction:	O O O O	✗: SL
Reference:	B-CHEM O B-CHEM O	✓: B, F
	*episodios de NF* (‘episodes of FN’ [‘febrile neutropenia’])	
Prediction:	O O O	✗: B, SL
Reference:	O O B-DISO	✓: F
FPs	*gen AVXS-101* (‘AVXS-101 gene’)	
Prediction:	O B-CHEM	✗: B, SL
Reference:	O O	✓: F
	*estudio BREATH-19* (‘BREATH-19 study’)	
Prediction:	O B-DISO	✗: SL
Reference:	O O	✓: B, F
Scope	*eventos adversos graves* (‘severe adverse events’)	
Prediction:	B-DISO I-DISO I-DISO	✗: B, SL
Reference:	B-DISO I-DISO O	✓: F
	*cirugía ginecológica abierta* (‘open gynecologic surgery’)	
Prediction:	B-PROC I-PROC I-PROC	✗: B, F, SL
Reference:	B-PROC I-PROC O	
	*estudios comparativos de la eficacia* (‘compared efficacy studies’)	
Prediction:	B-PROC O O O O	✗: SL
Reference:	B-PROC I-PROC I-PROC I-PROC I-PROC	✓: B, F

Concerning this point, many errors arose in mentions of the type of study or trial (e.g. *estudio fase 3, aleatorizado, doble ciego*, ‘phase 3, randomized, double-blind study’). Besides the variability of the type of essay, many mentions include inside its scope some words that we did not annotate (e.g. the trial code or its duration).

Table 11 includes samples of the errors found (*FNs* stands for ‘false negatives’; and *FPs*, for ‘false positives’). Table 12 reports the average count (and standard deviation) of false positives and false negatives across semantic groups for the 10 evaluation rounds. We could not report these counts for BERT, because the evaluation library we used to evaluate it (Python seqeval) does not give these values.

**Table 12:** Average FPs and FNs (±standard deviation) per entity type (test set)

	SequenceLabeler		Flair	
	FPs	FNs	FPs	FNs
ANAT	112.20 (±34.74)	136.70 (±14.15)	80.20 (±9.34)	119.30 (±3.92)
CHEM	216.00 (±48.05)	267.00 (±28.87)	222.80 (±11.67)	198.80 (±5.37)
DISO	459.80 (±91.15)	494.50 (±122.08)	380.30 (±9.48)	369.70 (±7.06)
PROC	690.50 (±138.92)	648.40 (±34.86)	692.80 (±15.45)	610.40 (±12.45)

We analyzed the variation of the annotated terms across entity types, to shed light on the errors this might cause. Following [74], we examined the average number of tokens or characters in entities, or the presence of coordination, numerals, punctuation characters, uppercase or stop words (Table 13). DISO and PROC entities tend to be longer or have more tokens. This is due to the use of modifiers (*grave*, ‘severe’), which we observed to cause errors related to the scope of terms. Also, regarding the PROC label, many entities refer to long mentions of trial types. Coordination and stop words are also more frequent in these entity types: e.g. *terapia biológica u hormonal*, ‘hormonal and biological therapy’; *cancer de cabeza y cuello*, ‘head and neck cancer’). Other superficial characteristics such as numerals, uppercase or hyphens occur more often in CHEM entities (e.g. *PM01183, 5-FC, ABT-530*). These features cause false positives in the neural models. Names of genes or trial studies in uppercase or with numbers might be misrecognized also as CHEM entities; and hyphens might cause errors related to the tokenization of entities. Punctuation characters appear more in PROC entities; this is because we annotated long mentions of trial types with commas or brackets (*Ensayo clínico fase II, aleatorizado*, ‘Phase 3, Randomized, Study’; *ensayo clínico terapéutico (fase III)*, ‘therapeutic clinical trial (phase III)’). Punctuation characters might cause misrecognition errors related to tokenization. The systems seldom annotate commas or brackets (they are interpreted as entity boundaries). ANAT entities are shorter and do not show a high frequency of any of these features. The large number of errors in this label might rather be due to the fact that this entity type is the least common in our data (the neural models lack enough samples to learn).

**Table 13:** Analysis of annotated entities (mean ±standard deviation) per label

	ANAT	CHEM	DISO	PROC
Mean tokens	1.20 (±0.57)	1.33 (±0.91)	2.06 (±1.35)	2.20 (±1.83)
Mean characters	9.32 (±5.09)	11.53 (±6.88)	16.74 (±10.28)	18.31 (±13.55)
Coordination	0.28% (±5.31)	0.15% (±3.89)	2.38% (±15.24)	3.86% (±19.27)
Has hyphen	0.30% (±5.44)	4.66% (±21.08)	2.88% (±16.72)	2.04% (±14.12)
Has numerals	0.37% (±6.08)	6.97% (±25.47)	3.24% (±17.70)	2.13% (±14.45)
Has punctuation	0.03% (±1.72)	0.18% (±4.29)	0.80% (±8.89)	3.48% (±18.34)
Has stop words	1.72% (±13.02)	6.35% (±24.39)	18.33% (±38.69)	22.26% (±41.60)
Uppercase	3.67% (±18.81)	13.65% (±34.33)	10.55% (±30.73)	10.46% (±30.60)

## Discussion

As for the use case experiment, the BERT model fine-tuned in the NER task yielded better results; still, the Flair and SequenceLabeler frameworks performed competitively and did not require a heavy pre-training step. Flair tended to yield slightly higher recall (sensitivity) values, whereas BERT and SequenceLabeler showed moderately higher precision (positive predictive value). Our intuition is that using specific embeddings trained on data from EudraCT could presumably improve our outcomes. This is a line of work that deserves to be pursued. In particular, using data from the domain to train a Spanish medical BERT or medical Flair embeddings, similar to the BioBERT [75] or HunFlair models [76], respectively. Another limitation of our experiments is that we did not test other embedding representations such as ELMo [77] or pooled contextual string embeddings [78], which yielded outstanding results in recent works [79]. The systematic comparison of approaches to NER with this corpus is out of the scope of this article. Given the current fast increase in neural architectures, it would be better made in the context of an evaluation challenge. Testing hybrid architectures [80], which combine language modeling, lexicon-based annotation and rule-based pattern matching, is a line to explore.

The need for more annotated data and the nature of the task might also have an impact on the results reported here. We observed in our error analysis that recognizing entities in clinical trials might pose difficulties related to the high variability of contents or the mentions of investigational drugs, which occur at low frequency even in domain data. If labeled data are scarce, purely machine-learning-based models or neural-based approaches might need to be complemented with terminology-based or rule-based approaches and pattern matching. This is, however, an intuition to test empirically.

The results in our experiments might partially be explained by the type of entities considered. We acknowledge that annotating only four UMLS groups is a limitation. Not all UMLS groups were labeled owing to time limits and because this first annotated version was a *proof-of-concept* to assess the annotation and the NER results: we focused on entity types that seemed more adequate for the task. Because the experiments showed that the annotation scheme and methodology provided decent results, annotating finer entity types is worth considering. Widening the annotation to other UMLS groups for devices (DEVI), physiological processes (PHYS) or genes (GENE) would enrich the corpus. However, according to our experience, other UMLS semantic groups related to concepts (CONC) might cause noise. It would be rather more adequate to distinguish finer-grained concept categories that are not UMLS groups. Namely, for discriminating drug attributes (administration route, dosage, strength or concentration) and for time expressions (date, duration or frequency), as in other works [81]. Another limitation is the fact that we did not annotate negation cues (e.g. *no*, ‘not’, or sin, ‘*without*’). Finally, the corpus would benefit from annotating semantic relations between entities (e.g. diso affects anat, or chem treats diso).

Overall, the preliminary experiments conducted show that the current version of the CT-EBM-SP corpus can be applied to test a wide range of approaches to biomedical NER. Our resource opens a new research line for Spanish NLP in the clinical trials domain. The annotation, carried out by medical and terminology professionals, has produced quality data, as shown by the high inter-annotator agreement achieved. Even though this resource lacks a rich variety of entity types, we have shown that competitive results can be obtained at its current state. Our tests come along resources and code to replicate and generalize our preliminary outcomes.

Given that this corpus includes texts also available in English, if needed, parallel texts may be collected in the future. Similar documents or the same translated texts are available in PubMed, EudraCT or SciELO [82]. Therefore, similar corpora can be collected and annotated in other languages. This paves the way towards creating standard resources that enhance the replicability of research across languages.

## Conclusion

We have described the methods to create the CT-EBM-SP corpus, a collection of 1200 texts about clinical trials studies and announcements in Spanish. This is the first resource for medical natural language processing of clinical trials in this language. Three experts have annotated it with entities from the Unified Medical Language System^®^ semantic groups (ANAT, CHEM, DISO and PROC). A 10% of the corpus was doubly annotated and a high inter-annotator agreement was achieved (average F1 = 85.65% ±4.79, strict match; 93.94% ±3.31, relaxed match). We presented use case experiments to show that the current version of the CT-EBM-SP corpus allowed us testing state-of-the-art neural biomedical named entity recognizers with competitive results. The presented methods are generalizable to other languages such as English, French or German, for which similar sources are available.

We believe this work contributes to enhancing the access to evidence-based information for both health professionals and patients. We would also be very satisfied if this resource played a beneficial role for developing systems that help patients to understand trial protocols, interventions and procedures better.

## Supplementary information

**Additional file 1.** Graphical abstract.

**Additional file 2.** Video demonstration of the annotation tool to preannotate texts of clinical trials.

## Data Availability

All the resources supporting this article are available at the project website: http://www.lllf.uam.es/ESP/nlpmedterm_en.html. The corpus is available at: http://www.lllf.uam.es/ESP/nlpdata/wp2/CT-EBM-SP.zip. The final annotation guidelines are available at: http://www.lllf.uam.es/ESP/nlpdata/wp2/annot_guideline_nlpmedterm.pdf The Python notebook with the code for the replicability of results is available at: https://github.com/lcampillos/Medical-NER. The embeddings can be downloaded at: http://www.lllf.uam.es/ESP/nlpdata/wp2/word-embeddings.zip.

## Abbreviations

ADR: Adverse Drug Reactions
BERT: Bidirectional Encoder Representations from Transformers
Bi-LSTM: Bidirectional Long-Short Term Memory
BIO: Begin Inside, Out
BIOES: Begin, Inside, Out, End, Single
BioNLP: Biomedical Natural Language Processing
CRF: Conditional Random Fields
CT: Clinical Trials
CTA: Clinical Trials Announcement
CUI: Concept Unique Identifier
EBM: Evidence-Based Medicine
EHR: Electronic Health Record
EMA: European Medicines Agency
EudraCT: European Clinical Trials Register
FN: False Negative
FP: False Positive
IAA: Inter-Annotator Agreement
ICD-10: International Classification of Diseases, 10th edition
KL: Kullback-Leibler
M: Mean
MeSH: Medical Subject Headings
MLM: Masked Language Model
NER: Named Entity Recognition
NLP: Natural Language Processing
OOV: Out-of-Vocabulary
P: Precision
PIBOSO: Patients/Population, Interventions, Background, Outcome, Study Design, Other
PICO: Patients/Population, Interventions, Comparators and Outcomes
PoS: Part-of-Speech
R: Recall
RCT: Randomized Control Trials
REEC: Repositorio Español de Estudios Clínicos
SciELO: Scientific Library Online
SD: Standard Deviation
SG: Semantic Group
SNOMED CT: Systematized Nomenclature of Medicine Clinical Terms
SPC: Summary of Product Characteristics
TP: True Positive
UMLS^®^: Unified Medical Language System^®^.

## Footnotes

1 http://www.lllf.uam.es/ESP/nlpdata/wp2/annot_guideline_nlpmedterm.pdf.
