# Correction to: A clinical trials corpus annotated with UMLS entities to enhance the access to evidence‑based medicine

**DOI:** 10.1186/s12911-021-01475-0

**Published:** 2021-04-07

**Authors:** Leonardo Campillos-Llanos, Ana Valverde-Mateos, Adrián Capllonch-Carrión, Antonio Moreno-Sandoval

**Affiliations:** 1 Computational Linguistics Laboratory, Universidad Autónoma de Madrid, C/Francisco Tomás y Valiente 1. Cantoblanco Campus, 28049 Madrid, Spain; 2 Medical Terminology Unit, Spanish Royal Academy of Medicine, C/Arrieta 12, 28013 Madrid, Spain; 3 Complejo Asistencial Hospital Benito Menni, C/Jardines 1, 28350 Ciempozuelos, Madrid, Spain

## Correction to: BMC Med Inform Decis Mak 21, 69 (2021) https://doi.org/10.1186/s12911-021-01395-z

Following publication of the original article [1], several errors were reported in the “Evaluation procedure” section, and in Table 4 and Table 8.

In the “Evaluation procedure” the error concerned the first two equations, and the incorrect and correct versions are given below:

$P= \frac{TP+FP}{TP}$ has been corrected to $P= \frac{TP}{TP+FP}$

$R= \frac{TP+FN}{TP}$ has been corrected to $R= \frac{TP}{TP+FN}$

In Table 4 the value for the total number of Annotated sentences, 14,051, was missing and has now been inserted.

In Table 8 the missing value for the ‘Test’ column for the row of ‘All’ Tokens, 58,300, was misplaced in the ‘Results of the experiments’ section. This has now been placed in the correct cell.

The original article has been updated.
