# Supplementary figures and images for: A clinical trials corpus annotated with UMLS entities to enhance the access to evidence-based medicine
Source: BMC Med Inform Decis Mak. 2021 Feb 22;21:69. doi: 10.1186/s12911-021-01395-z (PMC7898014; doi:10.1186/s12911-021-01395-z)

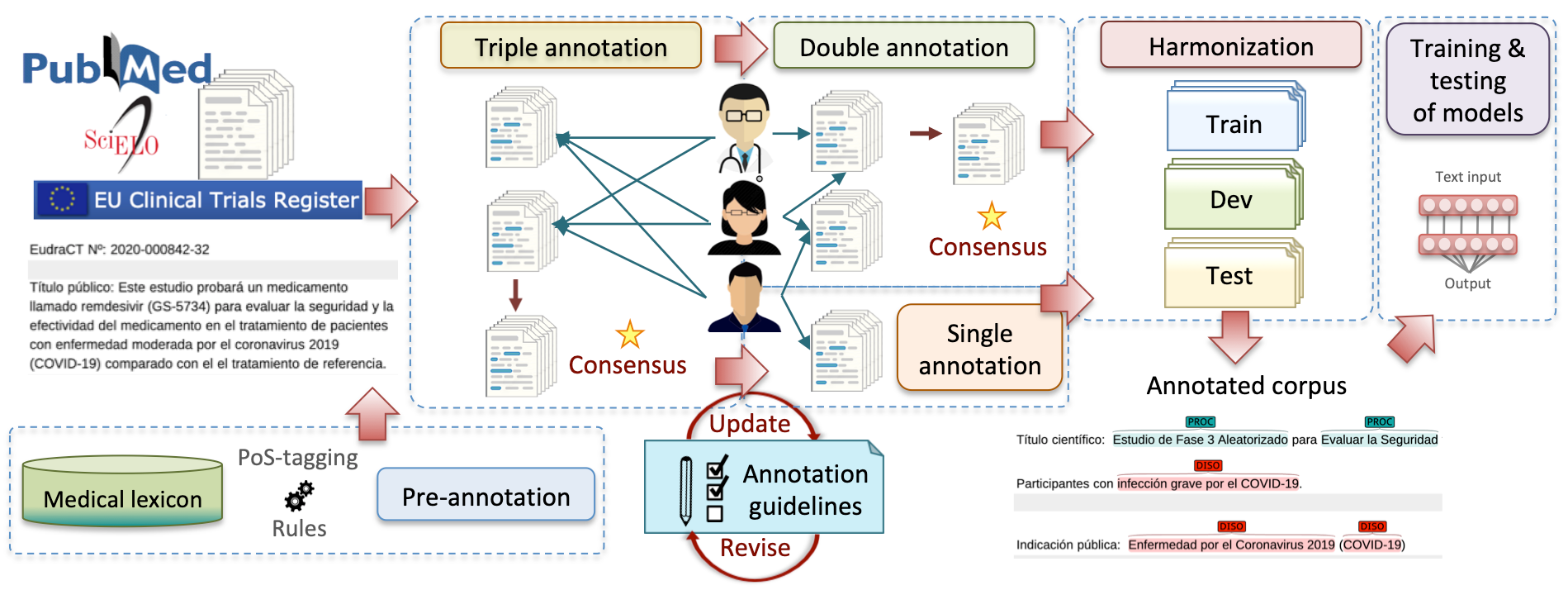

Supplement: Supplementary file 1 — Additional file 1. Graphical abstract. [file 12911_2021_1395_MOESM1_ESM.png]
